# Pre- and Post-treatment Impact of Antioxidants on Astrocytic Reactive Oxygen Species—An Undergraduate Classroom Research Project

**DOI:** 10.1021/acsomega.5c07652

**Published:** 2025-11-06

**Authors:** Haley Yost, Kaejaren C.N. Caldwell, Jooyoung Cho, Lindsay E. Cormier, Tanea T. Reed

**Affiliations:** † 6885 Eastern Kentucky University, 521 Lancaster Ave, Richmond, Kentucky 40475, United States; ‡ 2514 University of Cincinnati, 312 College Dr, Cincinnati, Ohio 45221, United States

## Abstract

Undergraduate research is a method to enhance student engagement in science courses as well as expand scientific curiosity. One method in doing so is performing course undergraduate research experiences. This project started as an undergraduate research project and expanded into a six-week in-class honor’s research experience. The field of traumatic brain injury (TBI) evokes instant name recognition and interest among undergraduate students based on their previous knowledge of sports and related concussion consequences in the news and on social media platforms. Currently, there is no clear treatment for traumatic brain injury. The rise of TBI-related deaths and debilitation has driven research efforts. Secondary effects of TBI are a current focus for research efforts including the regulation of oxidative stress within mitochondrial pathways. This work aims to investigate antioxidant therapies in preventing oxidative stress, a consequence of traumatic brain injury. Oxidative stress was stimulated by hydrogen peroxide (H_2_O_2_) and *tert*-butyl hydrogen peroxide (t-BHP) in primary cortical rat astrocytes. Astrocytes received an antioxidant pretreatment or post-treatment by administering gamma-glutamylcysteine ethyl ester (GCEE), a glutathione precursor, in an effort to combat oxidative stress that triggers apoptosis. The results showed that GCEE had a statistically significant therapeutic effect as both a post-treatment and a pretreatment, but to a lesser extent. “After these positive initial results with GCEE, this project was expanded into an undergraduate honors course, where students selected additional antioxidant molecules to test. Although results varied from this student experience, it should be noted that this was the first research endeavor for 100% of the students involved, broadening their participation in research and bridging the gap between basic classroom concepts and translational research that could potentially add to our knowledge base of TBI therapeutic strategies.

## Graphical abstract

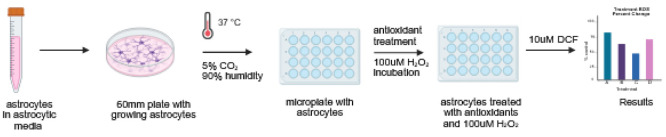

## Introduction

Eastern Kentucky University is an Appalachian serving institution, in which the service region includes twenty-two counties, twenty-one of which are considered Appalachian by the Appalachian Research Commission.^1^ As such over 30% are considered first generation students, therefore access to higher education has been limited. Authentic research in a classroom setting is ideal for this student population as it gives ownership to the student and allows them to have a higher level of engagement in the research planning, execution, and process. In regard to topics of study for a traditional research project, the topic of traumatic brain injury was selected based on recent events of interest to the students (i.e., professional athletes suffering from concussions, personal experience with the topic, etc.).

There are approximately 2.5 million traumatic brain injury (TBI) emergency department visits and 56,000 deaths, showing a 47% increase since 2007.^2^ Despite TBI being an increasing issue, there is no clear treatment strategy. TBI is primarily caused by accidents or falls so there are few preventive measures that can be taken. This differs from other leading medical diseases in America, such as heart disease and cancer where a person can alter their habits to decrease their chances of being affected. Symptoms can be mild, moderate, or severe depending on extent of brain damage. TBI severity is diagnosed based on the Glasgow Coma scale which assesses the level of cognition visually, verbally, and via motor skills. Primary stage traumatic brain injury is defined as initial effects of direct impact or damage to the brain tissue, impaired cerebral blood flow, and metabolic interference.^3^ Secondary stage TBI is classified as additional cell death due to depolarization of cell membranes that activate apoptotic pathways. Consequences of secondary TBI are oxidative stress, inflammation, and edema. Oxidative stress is the phenomenon by which there is an imbalance of oxidants and antioxidants in the body. Increases in oxidative stress have been well established and documented in neurodegenerative disorders such as Alzheimer’s disease, Parkinson’s disease, and Huntington’s disease.^4^ Researchers are targeting secondary stage TBI mechanisms such as brain edema, excitotoxic, and mitochondrial pathways. One potential target is antioxidant therapy to reduce levels of oxidative stress.^5–8^ Mitochondria house and consume vast amounts of reactive oxygen species (ROS). Reactive oxygen species are produced as a product of the electron transport chain and can have toxic cellular effects if remaining in their free radical state. Increased reactive oxygen/nitrogen species result in an elevation of oxidative stress. Mitochondrial glutathione, among other antioxidants and enzymes, works to maintain a healthy redox environment within the cell. By acting as a cofactor for glutathione peroxidase and glutathione-S-transferase, glutathione can oppose the damaging effects of hydrogen peroxide and lipid hydroperoxides.^9^

In this work, we explored the potential for a glutathione mimetic to act as a therapeutic agent for secondary TBI specifically comparing pre and post-treatment strategies. Gamma-glutamylcysteine ethyl ester (GCEE), as shown in Figure 1, is a precursor for glutathione was used in an effort to increase glutathione levels, thus counteract stimulated oxidant activity, decreasing oxidative stress within the cells. Apoptosis, caused by oxidative stress or ROS, is expected to then decrease or completely halt.

**Figure 1:**
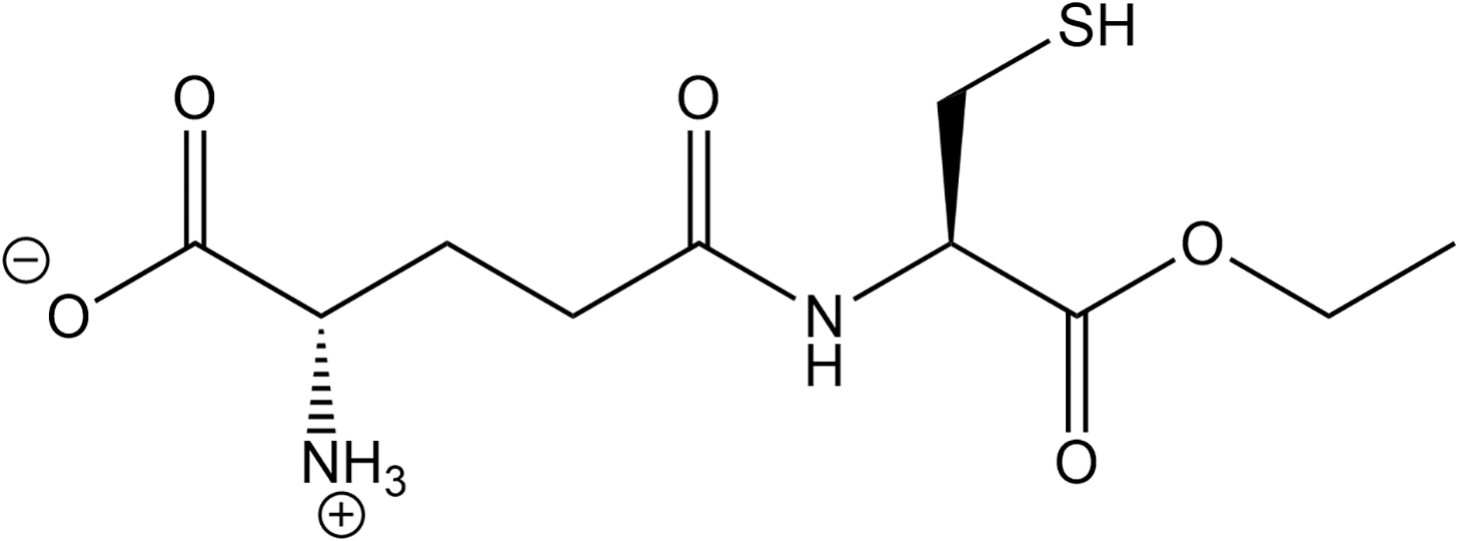
Structure of gamma-glutamylcysteine ethyl ester (GCEE), a glutathione mimetic.

In order to measure the oxidative stress a dichlorofluorescein (DCF) assay was performed. 2′,7′-dichlorofluorescein diacetate (H_2_DCFDA) is a probe that is de-esterified by esterases in the cell membrane. The non fluorescent product, 2“,7”-dichlorodihydrofluorescein (H_2_DCF) is converted to fluorescent dichlorofluorescein (DCF) upon interaction with a reactive oxygen species (i.e., H_2_O_2_ and t-BHP) as shown in Figure 2.

**Figure 2:**
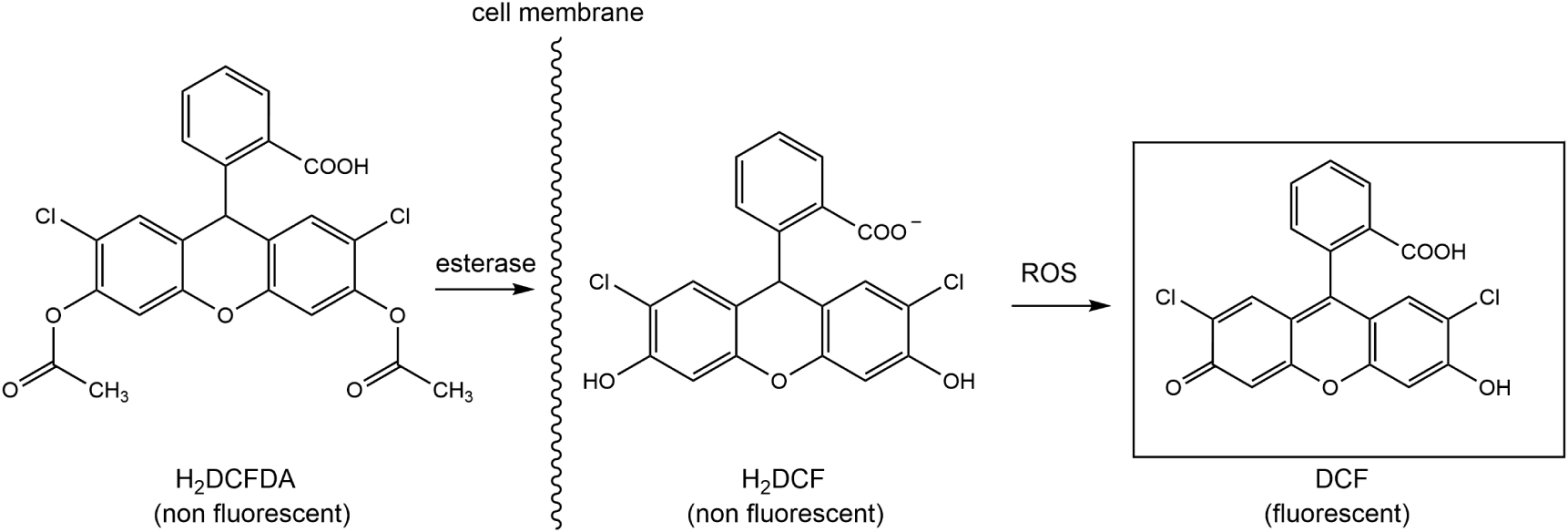
Reaction of the dichlorofluorescein (DCF) assay.

The positive outcomes from this work led to an expansion of this project in an honor’s classroom project. Sixteen students were enrolled in the course and divided into four groups of four. Although the cell culture procedures were identical, they were responsible for searching the scientific literature to choose a viable, cost-effective antioxidant therapy (<$50 per antioxidant) and developing a treatment plan for oxidative stress based on scientific literature. The antioxidants selected were cytidine 5′-diphosphocholine (citicoline), 5-methoxy-*N*-acetyltryptamine (melatonin), methyl 3,4-dihydroxybenzoate (MDBH), and quercetin. Dosage concentrations and conditions (pretreatment vs post-treatment) were chosen based on scientific literature. This provided students with critical thinking skills to improve their scientific literacy, establish a realistic protocol, and receive real world exposure to medicinal chemistry, biomedical science, and medicine as health costs are constantly rising more cost-effective treatments are needed.

### Cytidine 5′-Diphosphocholine (Citicoline)

Cytidine 5′-diphosphocholine (citicoline) is an intermediate in the phosphatidylcholine synthesis pathway. The structure is shown in Figure 3. The production of phosphatidylcholine is critical to healthy cell formation. Citicoline supplementation has been shown to increase dendritic length in early life^10^ and protect against age related memory deficits.^11^ Due to its positive outcomes in cognition, cytidine 5′-diphosphocholine has been evaluated in studies involving dementia and neurodegenerative disorders.^12–14^ Results are encouraging however; most studies use a combined therapy for treatment. The Citicoline Brain Injury Treatment (COBRIT) study examined the effects of daily oral citicoline administration on patients who sustained a traumatic brain injury. Findings demonstrated that the use of citicoline post injury did not significantly improve function and cognition in TBI patients.^15^ This work evaluated the treatment with citicoline post oxidative insult making the results novel.

**Figure 3:**
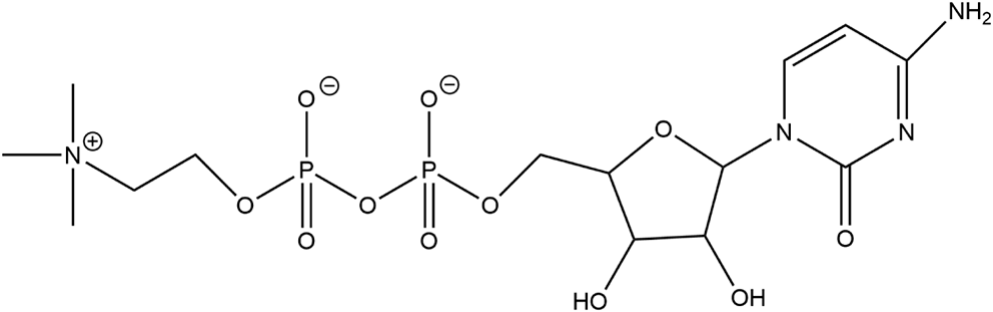
Structure of cytidine 5′-diphosphocholine.

### Melatonin

Melatonin is a hormone responsible for regulating sleep. It reaches peak levels at night, therefore getting the proper amount of sleep on a daily basis is necessary for its optimal activity. This hormone has been studied as an antioxidant as it scavenges reactive oxygen species, alleviates metal toxicity, boosts antioxidant activity, and prevents lipid peroxidation, another oxidative stress consequence.^6,16^ Melatonin has been found to reduce apoptosis and inflammation, both sequelae of TBI.^17^ It is reasonably priced and easily available in markets and retail stores, making it a feasible strategy. The use of melatonin has been studied in neurodegenerative diseases such as Alzheimer’s and Parkinson’s disease.^18^ This work will confirm the previous studies investigating pre and postinjury treatment of traumatic brain injury using melatonin.^19^ The structure for melatonin is shown in Figure 4.

**Figure 4:**
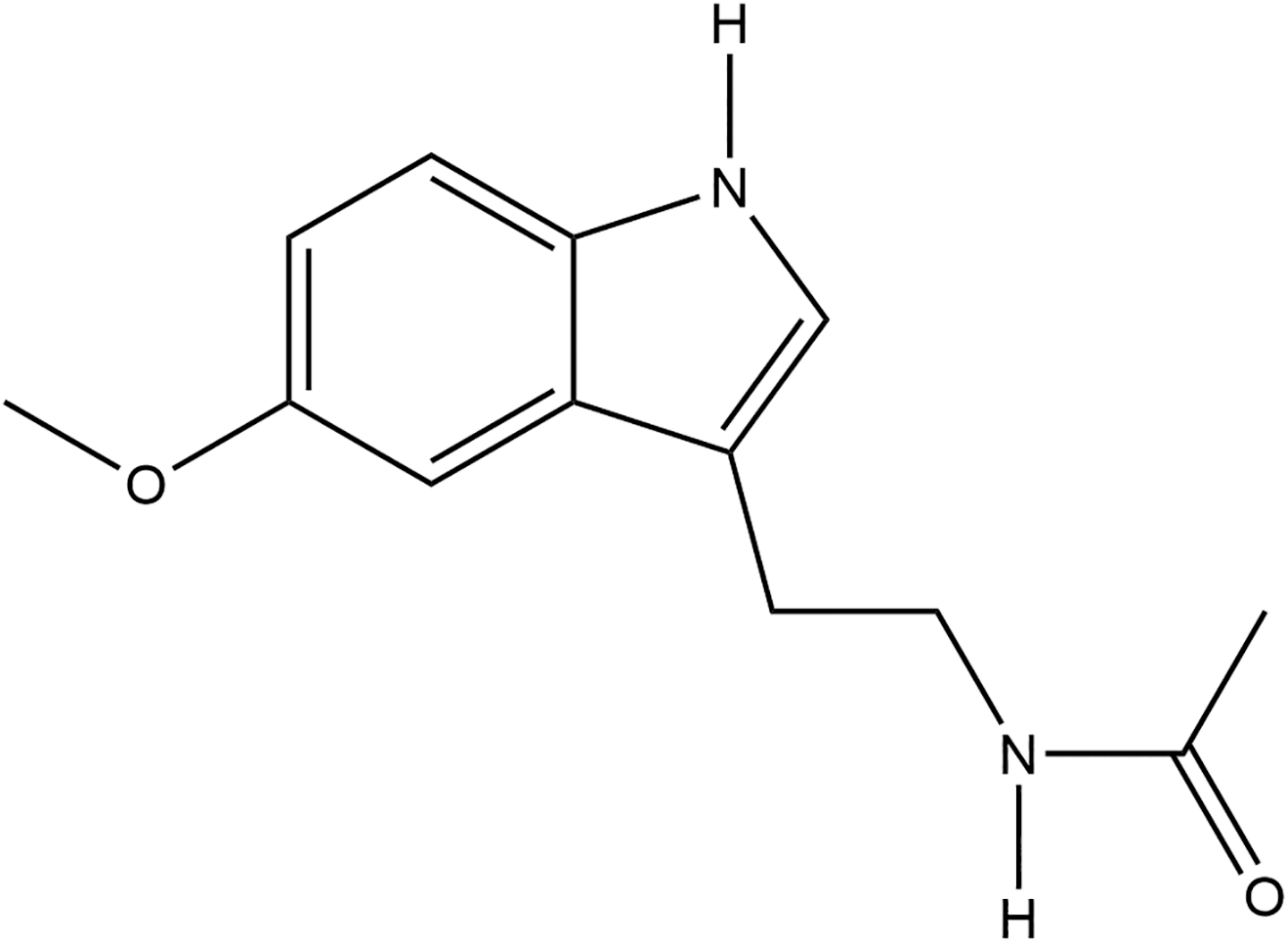
Structure of melatonin.

### Methyl 3,4-Dihydroxybenzoate

Methyl 3,4-dihydroxybenzoate (MDHB) is the methyl ester of 3,4-dihydroxybenzoic acid, a metabolite found in polyphenolics from green tea, which is rich in antioxidants. It has been shown to be both an antioxidant and a neuroprotectant against oxidative damage by upregulating the Nrf2 pathway.^20,21^ This compound has been shown to protect against amyloid beta toxicity associated in Alzheimer’s disease after 24 h.^22^ Through its strong potential as an antioxidant capacity, this compound was selected for investigation. Although, MDHB is not readily available as a commercial therapeutic treatment, the students felt that this compound was worthy of study (Figure 5).

**Figure 5:**
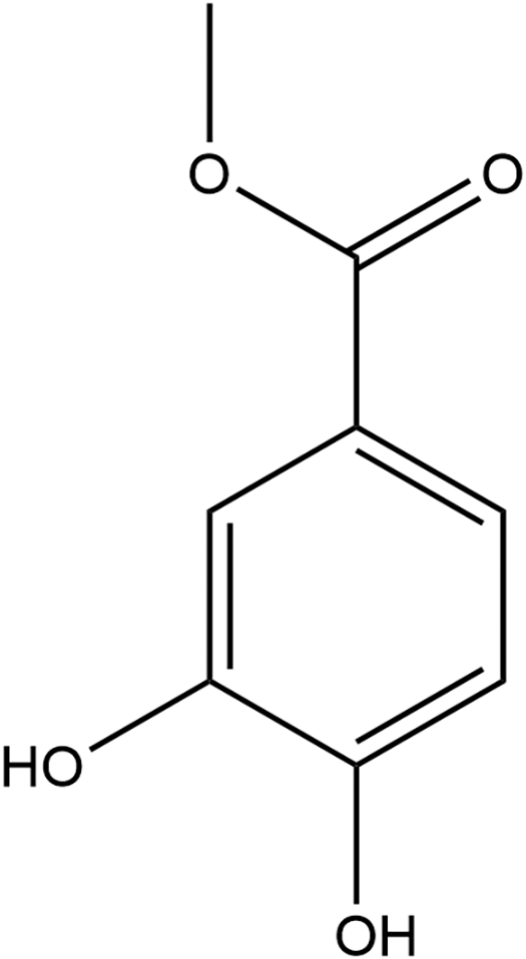
Structure of methyl 3,4-dihydroxybenzoate.

### Quercetin

Quercetin is a plant flavonol abundantly found in fruits and vegetables. Apples, grapes, kale, onions, berries, tomatoes, onions, and lettuce yield the highest amounts of quercetin. This antioxidant has been found to increase glutathione levels further increasing antioxidant capacity.^23,24^ This antioxidant has multiple health benefits including inhibition of ROS, prevention of inflammation, and antibacterial properties. Although not a cure, studies have shown that quercetin may have potential use as a treatment strategy for Alzheimer’s disease based on its ability to lower β secretase activity which reduces amyloid beta peptide production, a common feature observed in this neurological disorder.^25,26^ Quercetin has been studied as a treatment for mild TBI post injury, however this work will investigate the use of quercetin as a pretreatment therapy.^27^ As quercetin is readily available in nutritional form, it can be easily accessible as a feasible treatment (Figure 6).

**Figure 6:**
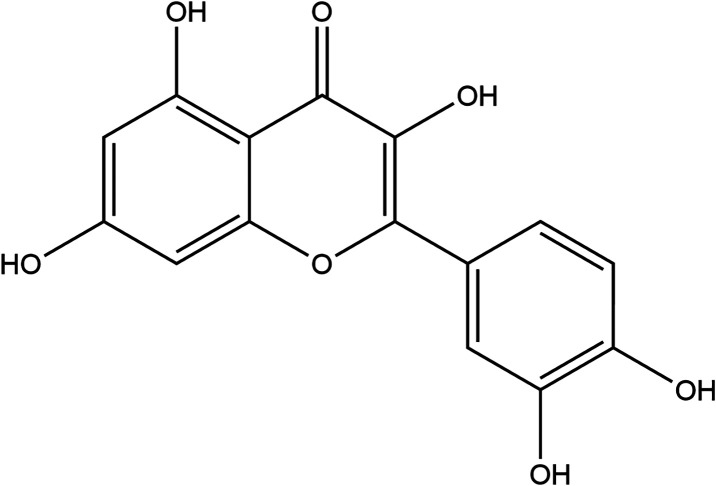
Structure of quercetin.

## Results and Discussion

### GCEE Results

Control (DMSO) showed no change in cell death. There was a statistically significant increase in ROS production in cells incubated with H_2_O_2_ and t-BHP (Figure 7). The increases were 50% and 27%, respectively. ROS levels were decreased via GCEE treatment for both oxidative insults (40%, H_2_O_2_ + GCEE and 15% t-BHP + GCEE). These findings were statistically significant. Post-treatment with GCEE yielded positive results as ROS levels were reversed upon GCEE administration. Results from post-treatment showed greater improvement in oxidative stress levels with both H_2_O_2_ and t-BHP (Figure 8). Measurable statistically significant increases in ROS production were observed in H_2_O_2_ and t-BHP treatment. ROS levels were decreased via GCEE treatment for both oxidative insults (65%, H_2_O_2_ + GCEE and 30% t-BHP + GCEE). These findings were statistically significant.

**Figure 7:**
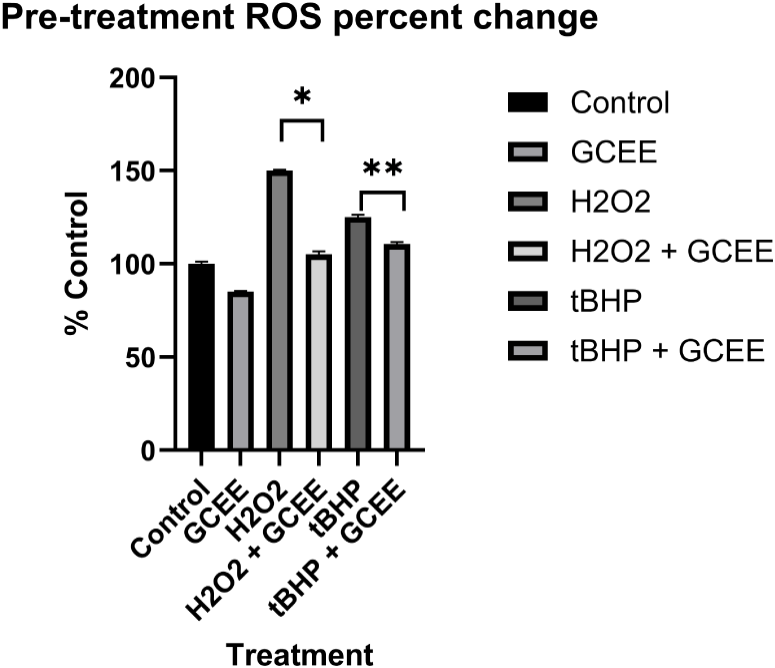
Pretreatment group percent of ROS compared to control. GCEE showed significant reductions in ROS levels from H_2_O_2_ (*p < 0.00002) and t-BHP (**p < 0.04) stimulated oxidative stress. Error is reported in SEM.

**Figure 8:**
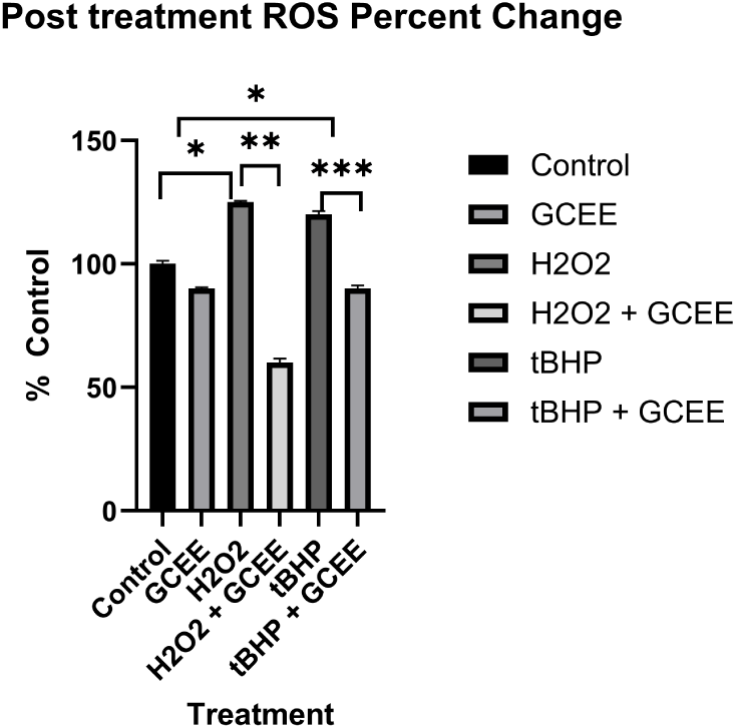
Post-treatment group percent of ROS compared to control. Oxidant insult by H_2_O_2_ and t-BHP were significantly increased (*p < 0.05). GCEE treatment reduced oxidative stress induced significantly by H_2_O_2_ (**p < 0.0002) and t-BHP (***p < 0.04). Error is reported in SEM.

### Antioxidant Results

Due to time and budgetary constraints, only hydrogen peroxide was used as an oxidant in the classroom project. Control (DMSO) showed no change in cell death. There was a statistically significant increase in ROS production in cells incubated with H_2_O_2_ (Figure 9). The increase was 280%. ROS levels were decreased via antioxidant treatment (56%, H_2_O_2_ + melatonin, 65% H_2_O_2_ + quercetin, and 33% H_2_O_2_ + MDHB). These findings were statistically significant. Post-treatment with citicoline yielded positive results as ROS levels (Figure 10). ROS levels were decreased by 72% via citicoline treatment. These findings were statistically significant. It should be noted that citicoline was the only antioxidant chosen for a post-treatment based on literature.^15^

**Figure 9:**
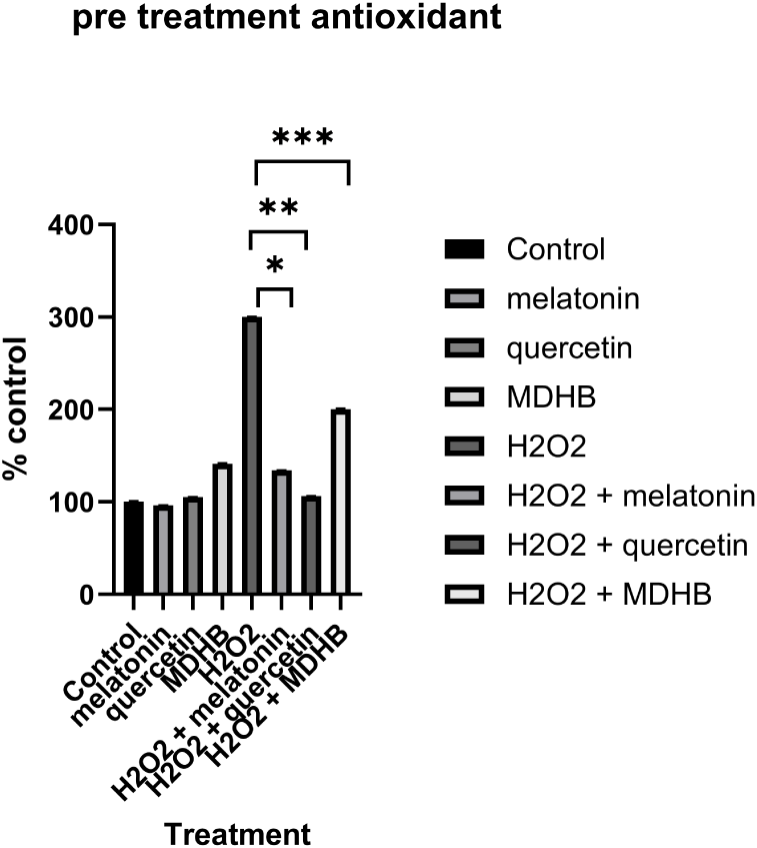
Pretreatment group percent of ROS compared to control. Melatonin, quercetin, and MDHB showed significant reductions in ROS production as induced by H_2_O_2_ (*p < 0.0001, **p < 0.02, and **p < 0.05). Error is reported in SEM.

**Figure 10:**
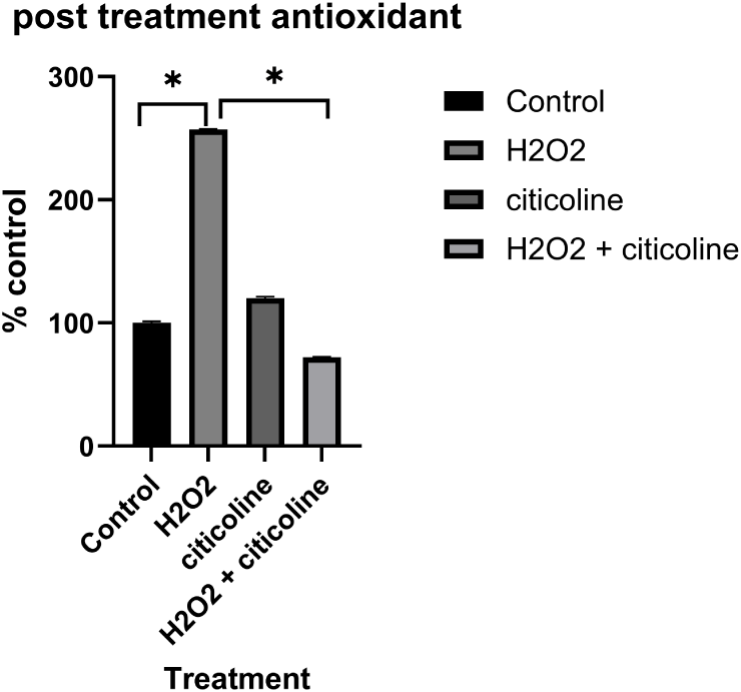
Post-treatment group percent of ROS compared to control. Oxidant insult by H_2_O_2_ was significantly increased (*p < 0.05). Citicoline treatment reduced oxidative stress induced significantly by H_2_O_2_ (*p < 0.05). Error is reported in SEM.

## Conclusions

In conclusion, our experiment bolsters supporting evidence that the antioxidant GCEE has potential to be used to reduce apoptosis due to oxidative stress in TBI. From a statistical perspective, our results showed that GCEE post-treatment is more effective when compared to GCEE treatment following oxidative stress. The pretreatment results showed that GCEE can significantly reduce oxidative stress induced by H_2_O_2_ (*p* < 0.00002) and to a lesser extent t-BHP (*p* < 0.04). The post-treatment group showed that GCEE can reduce oxidative stress for H_2_O_2_ and t-BHP but was shown to be significant at each treatment. Reductions in ROS levels were observed in the post therapy strategy compared to the pretreatment strategy. This is applicable to taking medication after post incident. The classroom antioxidant experiment validated previous findings regarding TBI treatments on a smaller scale. All finds were statistically significant in regard to oxidant versus treatment. Students were able to develop a scientific hypothesis, establish a protocol, add to their laboratory skill set (i.e., pipetting and preparing solutions), learn data analysis, and present their research findings to the class. This experience sets the foundation for expansion as additional, cost-effective strategies may be evaluated. This work could translate into more effective TBI treatment options. Overall, this classroom undergraduate research experience was a success as it provided a creative way to broaden participation in research, pique student interest, and extend their fundamental knowledge of biochemistry into real world applications.

## Methods

### Cell Culture

Primary cortical rat astrocytes were obtained from Thermo Fisher.^28^ A 15 mL centrifuge tube was prerinsed with the astrocyte media (85% Dulbecco’s Modified Eagle serum (DMEM), 15% Fetal Bovine Serum (FBS)) in a sterile laminar flow hood. The frozen one milliliter (1 × 10^6^ cells) vial was placed in a 37 °C water bath. Contents were gently swirled every 30 s until the thawed. The 15 mL centrifuge tube was rinsed for a second time with the media and the media was then discarded. The cells and astrocytic media-DMSO freezing solution within the vial were transferred to the prerinsed 15 mL tube. One milliliter of growth media was then slowly added to the vial to rinse the sides, then added to the 15 mL tube dropwise, avoiding bubble formation. An additional 8 mL growth media was added dropwise to the tube. The cell containing tube was centrifuged for 5 min at 300*g*. The supernatant was discarded, and cells were resuspended in 2 mL of growth media. A 60 mm cell plate was coated with 6.5 mL of growth media. The resuspended cells were then added evenly across the 60 mm plate in a dropwise fashion. The plate was gently rocked back and forth and then incubated at 37 °C, 5% CO_2_ and 90% humidity.

Once the astrocytes reached 100% confluence, they were ready to be passaged. Astrocyte media (85% DMEM, 15% FBS) and Accutase cell dissociation agent were prewarmed in a 37 °C water bath. Growth media was removed from the 60 mm astrocyte containing plate a milliliter at a time to avoid disrupting the cells and placed in a sterile 15 mL centrifuge tube. Approximately 6 mL of D-PBS (without Ca^2+^ and Mg^2+^) were added to the 60 mm cell plate and gently rocked back and forth. The D-PBS was then aspirated off and discarded. Cell detachment was initiated by adding 3 mL of the prewarmed Accutase to the cell plate and gently rocking the plate. The plate was then incubated for 20 min at 37 °C, 5% CO_2_, and 90% humidity, rocking the plate every 5 min. Growth media that was initially removed from the 60 mm plate and set aside is now added back to the Accutase-containing plate. All of the cell plate contents were transferred to a new, sterile 15 mL conical tube and centrifuged for 5 min at 300*g*. The supernatant was aspirated and discarded. The pellet was resuspended in 4 mL of growth media. Two new 60 mm cell plates were each coated with 4 mL of growth media. Each cell plate then received 2 mL of the cell, media mix from the 15 mL conical tube dropwise, evenly dispersed fashion. Both plates were then incubated at 37 °C, 5% CO_2_, and 90% humidity until reaching confluence. At that point, cells were then placed into a vial to be stored in liquid nitrogen until needed.

### Reactive Oxygen Species Assay

The frozen vial of cells was thawed, and contents were added to a 15 mL conical tube that was prerinsed with DMEM. The vial was then rinsed with DMEM to obtain any remaining cells to be transferred. The 15 mL tube was then centrifuged for 5 min a 300*g*. A hemostat was used to estimate the number of cells contained in one microliter of media-cell mixture. Astrocyte media (200 μL) was added to each well that would contain cells of a black bottom 90-well plate. Astrocytes were then seeded at 1.2 × 10^4^ cells/well. The plate was then incubated for at least 24 h at 37 °C, 5% CO_2_, and 90% humidity to allow cells to adhere to the well surface. One side of the well plate contained the antioxidant pretreatment analysis and the other a post-treatment analysis. Each analysis contained a control group (serum free DMEM), GCEE, H_2_O_2_, GCEE + H_2_O_2_, t-BHP, and GCEE+ t-BHP. Astrocyte media was replaced with 100 μL serum free media in each well. To maintain a consistent well volume, serum free media was added to well with that did not receive GCEE treatment. The pretreatment treatment groups were treated with GCEE (750 mM) for 2 h at 37 °C, 5% CO_2_. Designated cells (H_2_O_2_, GCEE+ H_2_O_2_ t-BHP, and GCEE+ t-BHP) were treated for 18 h with either H_2_O_2_ (100 μM) or t-BHP (100 μM) at 37 °C, 5% CO_2_ for a total treatment period of 20 h. The post-treatment groups were first treated for 18 h with the oxidant; either H_2_O_2_ (100 μM) or t-BHP (100 μM) at 37 °C, 5% CO_2_. Then, the cells were treated with GCEE (750 μM) or serum free media for 2 h at 37 °C, 5% CO_2_ for a total treatment period of 20 h. For antioxidant studies the concentrations and conditions were based on current literature. Pretreatment groups were treated with melatonin (200 μM), MDHB (20 μM), and 10 mM of quercetin 24 h before adding 100 μM H_2_O_2_ to induce oxidative stress. The post-treatment group (citicoline, 100 μM) was incubated in media 24 h before 100 μM H_2_O_2_ was added. Due to student and instructor schedules, 24 h was the best time point to complete this work. All other procedures regarding cell culture were identical. Upon conclusion of the treatment period, all the cells were rinsed twice with sterile water. Following the rinses, 2,7-dichlorodihydrofluorescein diacetate (10 μM) solubilized in PBS was added to the cells and incubated for 30 min at 37 °C, 5% CO_2_. The plate was then read by a spectrophotometer for 35 min, collecting values every 2.5 min with an emission wavelength of 485 nm and excitation of 528 nm.

### Data Analysis

All statistical analyses were performed using GraphPad PRISM software and Student’s *t* test. Significance of each result was confirmed by calculation of the *p* value by two-tailed Student’s *t* test (*p* values <0.05). Error bars were calculated as SEM.
